# Virtually Augmented Self-Hypnosis applied to endovascular interventions (VA-HYPO): Randomized Controlled Trial Protocol

**DOI:** 10.1371/journal.pone.0263002

**Published:** 2022-02-23

**Authors:** Salah D. Qanadli, Louis Gudmundsson, Giuseppe Gullo, Alexandre Ponti, Sarah Saltiel, Anne-Marie Jouannic, Mohamed Faouzi, David C. Rotzinger

**Affiliations:** 1 Cardiothoracic and Vascular Division, Department of Diagnostic and Interventional Radiology, Lausanne University Hospital and University of Lausanne, Lausanne, Switzerland; 2 Center for Primary Care and Public Health (Unisanté), University of Lausanne, Epalinges, Switzerland; Goethe University Frankfurt: Goethe-Universitat Frankfurt am Main, GERMANY

## Abstract

Endovascular interventions (EVI) are increasingly performed as minimally-invasive alternatives to surgery and have many advantages, including a decreased need for general anesthesia. However, EVI can be stressful for patients and often lead to anxiety and pain related to the procedure. The use of local anesthetics, anxiolytics, and analgesic drugs can help avoid general anesthesia. Nevertheless, these drugs have potential side effects. Alternative nonpharmacological therapies can improve patients’ experience during conscious interventions and reduce the need for additional medications. The added value of virtually augmented self-hypnosis (VA-HYPO) and its potential to reduce pain and anxiety during peripheral and visceral arterial and venous EVI is unknown. This is a prospective two-arm trial designed to randomize 100 patients in two groups according to the use or not of VA-HYPO during peripheral EVI as a complementary nonpharmacological technique to improve patient comfort. The main objective is to compare per-procedural anxiety, and the secondary aim is to compare the rated per-procedural pain in both groups. The potential significance is that VA-HYPO may improve patients’ experience during peripheral and visceral arterial and venous EVI and other minimally invasive interventions performed under local anesthesia.

**Trial registration**: Our study is registered on clinicaltrials.gov, with trial registration number: NCT04561596.

## Introduction

Endovascular interventions (EVI) are minimally invasive therapies gaining interest in many indications. EVI are increasingly replacing surgery and vastly reduce the need for general anesthesia. Indications for percutaneous interventions are many and varied; typical examples include treatments for venous disorders [1], aneurysms [2], and peripheral and visceral arterial diseases [3]. Interventions whose sole purpose is to gain vascular access, such as for peripherally inserted central catheters, are performed exclusively under local anesthesia [4]. Furthermore, outpatient procedures have been developed and expanded during the last decade [5]. Despite efforts regarding patient information, preparation, and management for EVI, patients often experience periprocedural apprehension, generating anxiety. During peripheral EVI–procedures primarily performed under local anesthesia–pain feelings at various levels are observed. Such states are not specific to EVI and have frequently been reported in patients admitted for cardiovascular surgery, ranging from 20% to 60% [6, 7]. Nonetheless, they might be exacerbated by the environment required for EVI, which includes complex facilities (X-ray and hybrid interventional suites), advanced devices and technologies. Associated uncontrolled overcommunication on social media and free websites by non-healthcare professionals, whose quality is variable, might induce ambiguity and inconsistency, leading to anxiety [8]. Furthermore, preoperative anxiety significantly influences postoperative pain and patient outcomes [9, 10], particularly because anxiety and pain interact synergistically.

Periprocedural anxiety and pain are most commonly managed with rapid-acting opioid analgesics and/or hypnotic sedative drugs [11–14]. With minimal (Level 1 of the American Society of Anesthesiology [15]) and moderate sedation/analgesia (Level 2), spontaneous ventilation and cardiovascular function are usually maintained. In contrast, more profound sedation (Level 3) might require intervention for adequate ventilation. Despite appropriate delivery of drugs and careful monitoring, complications may occur. Complications include respiratory depression, hypotension, unconsciousness, paradoxical agitation, and nausea or vomiting. Preprocedural anxiety might be treated with anxiolytics. However, some drugs (e.g., Lorazepam) failed to decrease preoperative anxiety significantly [11].

Nonpharmacological pain and anxiety management approaches have been developed as adjunctive treatment [16–18]. Hypnosis induces a state of reduced peripheral awareness and can be used as a nonpharmacological intervention to optimize patients’ experience during EVI procedures, with significant analgesic and anxiolytic effects [19–23]. Dissociation is the main effect of hypnosis used during conscious medical interventions. One of the main limitations of using hypnosis is the human resources required, limiting the number of patients who can benefit from it. Virtually augmented self-hypnosis is a technique that combines the immersive and inductive effect of virtual reality, followed by suggestions for comfort and pain relief [24].

In experimental and clinical experience, virtual reality is considered a valuable tool to decrease anxiety and reduce pain by diverting attention from painful stimuli [25, 26]. It is interesting to notice that the effect on pain has been observed in both highly hypnotizable and less hypnotizable patients [27, 28]. However, available data regarding the advantages of using virtually augmented self-hypnosis (VA-HYPO) in clinical practice remains scarce, and its impact on pain and anxiety related to EVI is unknown.

## Materials and methods

### ● Aim, design, and setting of the study

This prospective study is based in a single Swiss academic hospital and seeks to evaluate whether virtually augmented self-hypnosis (VA-HYPO) lowers anxiety and pain in the setting of percutaneous peripheral and visceral arterial and venous EVI. VA-HYPO is performed using a head-mounted device (Oncomfort^TM^) providing a video screen and sound system aiming to achieve digital sedation. For this study, patients follow the "Aqua" module reproducing a scuba diving experience and breath exercises in a virtual reality environment. For this purpose, a monocentric open-label prospective randomized trial with two arms is designed.

### ● Sample size, inclusion, and exclusion criteria

A power analysis was conducted to determine a sample size that can detect a statistically significant anxiety decrease of at least 5 percent, such as encountered in a previous investigation using autohypnosis in the setting of systemic chemotherapy [29]. The Sample size calculation is based on the primary outcome "per procedural anxiety". The expected mean (sd) of per procedural anxiety in the control group is 45 (3) and 5% lower in the intervention group 42.75 (2). The minimum sample size needed to show that the mean difference is significant with a power of 90% and an alpha of 5% (two-sided) is 32 patients per group. To avoid loss of power due to possible dropout (20%), randomizing 40 patients in each group would be appropriate. Furthermore, because the primary outcome measure is patient-reported and subject to measurement error, we intend to add a safety margin and include 50 participants in each group since no additional risk nor financial burden is associated with study participation.

Inclusion criteria: participants will be patients subjected to interventional radiology for a peripheral vascular intervention under local anesthesia; participants will be over 18 years old and have signed an informed consent form. In this study, peripheral EVI refers to lower limb arterial angioplasty and stenting, renal and mesenteric angioplasty and stenting, venous interventions (vena cava filter placement, venous malformation embolization, peripherally inserted central catheter). Patients referred to cardiothoracic or neuroradiology intervention are not eligible.

Exclusion criteria: no or limited study language comprehension, deafness or visually impaired patient, the anticipated need for a sedative medication, a history of motion sickness or a psychiatric disease such as paranoia, schizophrenia, deep water phobia, and dementia. We will also exclude patients who do not tolerate the virtual reality mask during a preoperative visit.

### ● Characteristics of participants, randomization, and blinding

The sequentially numbered, opaque, sealed envelope (SNOSE) method was used for randomization with a 1:1 allocation before the intervention. A person not involved with patient recruiting will print the allocation sequence and prepare the envelopes (AMJ).

One group of participants will wear the virtual reality mask with the autohypnosis program running during the EVI. The other group will benefit from the intervention in standard conditions without the virtual reality mask.

The study will run in an open-label fashion because information cannot be withheld from trial participants and operators. Participants included in the virtual reality arm will wear the head-mounted device, and awareness regarding self-hypnosis is inevitable.

Study timetable: the first patient was included in August, 2020. The recruitment is expected to be completed by the end of May, 2022.

All vascular punctures are performed under local anesthesia and using ultrasound guidance. The puncture site depends on the clinical indication and is at the operator’s discretion. The typical vascular access is the common femoral artery for peripheral and visceral arterial interventions. The most common venous accesses are the common femoral vein and brachial veins.

Arterial vascular accesses usually benefit from vascular closer devices, at the operator’s discretion, while venous accesses are controlled by manual compression.

### ● Processes, interventions, and comparisons

**Fig 1:**
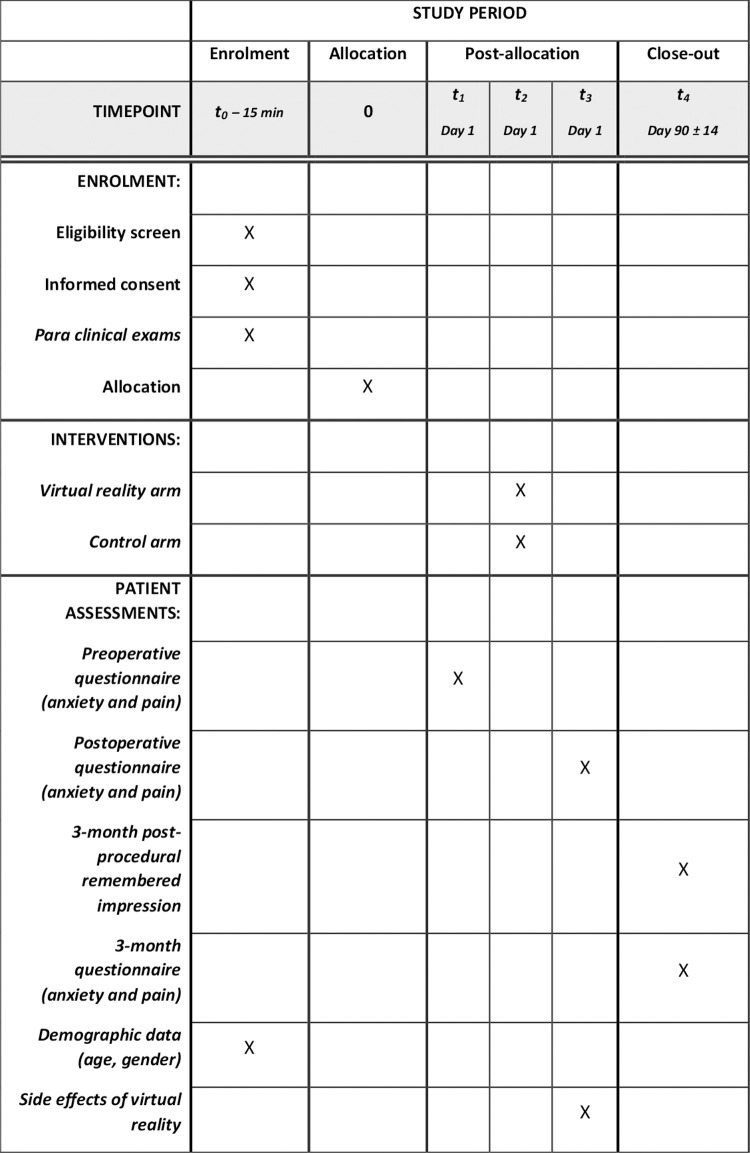
Schedule of enrolment, interventions, and assessments.

The schedule of enrolment, interventions, and assessment is presented in Fig 1. On the day of the intervention, after randomization, the participants will fill the operative anxiety self-questionnaire (Table 1) and indicate the intensity of pain felt on a segmented numerical version of the analog visual scale (Table 2). At the patient’s arrival in the operating room, an investigator will adapt the virtual reality mask if the patient is in the virtual reality group.

**Table 1:** Anxiety scale used before and after the endovascular intervention.

		Not at all	A little	Somewhat	Very much so
**1.**	I feel calm	**1**	**2**	**3**	**4**
**2.**	I feel secure	**1**	**2**	**3**	**4**
**3.**	I feel tense	**1**	**2**	**3**	**4**
**4.**	I feel strained	**1**	**2**	**3**	**4**
**5**	I feel at ease	**1**	**2**	**3**	**4**
**6.**	I feel upset	**1**	**2**	**3**	**4**
**7.**	I am worrying about possible misfortunes	**1**	**2**	**3**	**4**
**8.**	I feel satisfied	**1**	**2**	**3**	**4**
**9.**	I feel frightened	**1**	**2**	**3**	**4**
**10.**	I feel comfortable	**1**	**2**	**3**	**4**
**11.**	I feel self-confident	**1**	**2**	**3**	**4**
**12.**	I feel nervous	**1**	**2**	**3**	**4**
**13.**	I feel jittery	**1**	**2**	**3**	**4**
**14.**	I feel indecisive	**1**	**2**	**3**	**4**
**15.**	I am relaxed	**1**	**2**	**3**	**4**
**16.**	I feel content	**1**	**2**	**3**	**4**
**17.**	I am worried	**1**	**2**	**3**	**4**
**18.**	I feel confident	**1**	**2**	**3**	**4**
**19.**	I feel steady	**1**	**2**	**3**	**4**
**20.**	I feel pleasant	**1**	**2**	**3**	**4**

This questionnaire will be presented to participants in the French language, according to Gauthier et al. [30].

**Table 2:** Pain assessment scale used before and after the endovascular intervention.

Absence of pain	**1**	**2**	**3**	**4**	**5**	**6**	**7**	**8**	**9**	**10**	Worst pain imaginable

Participants will be asked to indicate the pain they currently feel on the pain scale (0 = absence of pain; 10 = worst pain imaginable).

The percutaneous EVI is performed as usual except for the autohypnosis software that will run during the intervention in the virtual reality group.

The medical team will provide pain and anxiolytic medication if needed during the intervention at the operator’s discretion and depending on the patient’s condition.

Immediately after the intervention, participants will fill a second self-questionnaire (postoperative questionnaire) to report anxiety and pain felt during the intervention (Tables 1 and 2).

A clinical visit at 3 months, as part of the standard clinical protocol, is planned for late assessment. Participants will fill a third self-questionnaire on anxiety (Table 1), the visual analog scale for pain (Table 2), and remembered per-procedural impressions (Table 3).

**Table 3:** Post-procedural remembered impressions.

Pleasant	**1**	**2**	**3**	**4**	**5**	**6**	**7**	**8**	**9**	**10**	Uncomfortable

At the 3-month visit, participants will be asked to indicate their current feelings related to the intervention on the following scale (0 = pleasant; 10 = uncomfortable)

If needed, would you be ready to repeat the experience?

Yes / Maybe / No.

**Fig 2:**
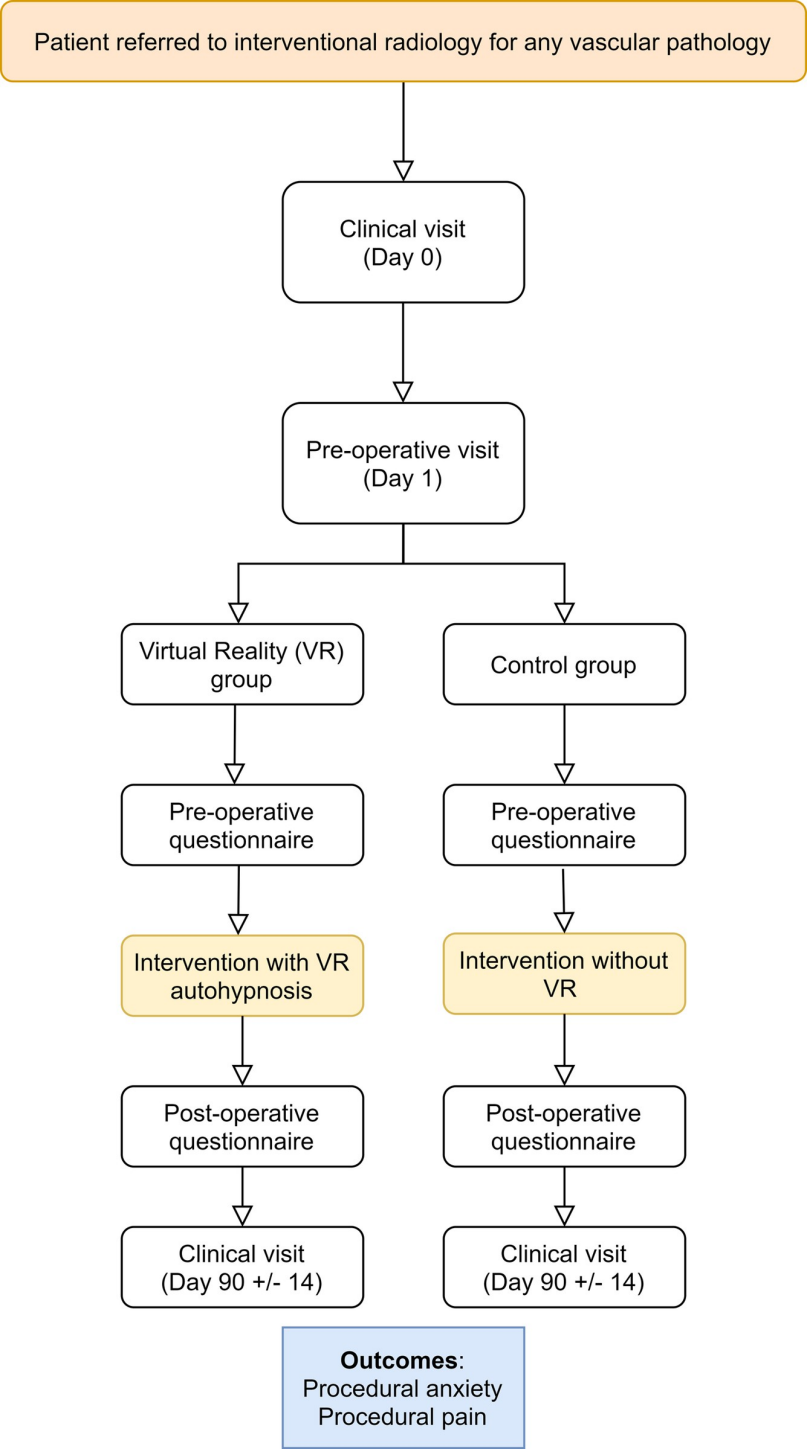
Study flowchart.

The study flowchart from referral to the last clinical visit is provided in Fig 2.

## ● Measured outcomes

The primary outcome is the rated per procedural anxiety, measured by the Spielberger Anxiety State Inventory in French [30]. The Spielberger Anxiety State Inventory is a self-questionnaire of 20 items, each item scores from 1 to 4. The test result is the sum of the 20 items; therefore, the results range from 20 to 80 (Table 1). A high score is related to a high level of anxiety at the moment of the test.

The secondary outcome is the rated per procedural pain measured by the visual analog pain scale. The visual analog pain scale rates pain from 0 to 10, 0 being no pain, and 10 being the worst pain imaginable (Table 2).

Both outcomes will be measured on day 1, before and after the intervention, and 3 months after the intervention.

The other outcomes of interest will be measured on day 1. Those outcomes are age and sex, side effects of virtual reality, the amount of drugs administered for pain and anxiety, the number of participants that did not tolerate the mask before randomization, and the number of participants where the mask has been taken off (patient decision/medical team decision). We will measure the prevalence of cybersickness, our safety outcome on day 1.

Patient-relevant outcomes, including amputation, mesenteric or renal ischemia, and death, will be reported 90 days after EVI.

### ● Data management

Data are coded and processed with Research Electronic Data Capture (REDCap), a broadly used HRA-compliant data collection application [31, 32]. Data will be archived in coded form for a minimum of 10 years after study completion.

### ● Safety considerations

To date, in the literature, the only recognized side effect of virtual reality is cybersickness [16], a condition similar to motion sickness but caused by immersion in virtual reality rather than by actual movement.

The prevalence of cybersickness is not known because of the diverse software and hardware that deliver virtual reality.

The manufacturer of the Oncomfort system also warns the users of the potential risks of:

- claustrophobia
- problems with orientation
- sweating of the face
- sleepiness

The prevalence of the above risks is not known.

The present study intends to compare vascular radiologic interventions with or without a virtual reality mask with a specific program to release stress and tension from patients undergoing certain procedures.

We will collect device deficiencies and adverse events, including all serious adverse events. Adverse events will be thoroughly investigated and documented in the medical record and the case report form (CRF) during the entire study period.

There is no follow-up period needed for this study since virtual reality has no known long-term adverse effect.

### ● Type of data and statistical analyses

Quantitative discrete data will be the Spielberger anxiety state self-questionnaire results, rated visual analog pain scale, age, the amount of drugs administered for pain and anxiety. We will compare the means of the discrete quantitative data with two-sided student tests.

Qualitative data are participants that did not tolerate the mask before randomization, participants that had to take off the mask, and the occurrence of cybersickness. We will compare the occurrence of qualitative data between both groups with the Wilcoxon signed-rank test.

### ● Ethical considerations and declarations

The study protocol (version 3, July 17th, 2020) received approval from the ethical committee of the Canton de Vaud (CER-VD) on July 27^th^, 2020 (protocol number 2020–00728). The study is compliant with the principles of the declaration of Helsinki and was registered on ClinicalTrials.gov (NCT04561596). Only patients who give written informed consent will be included. A data safety monitoring committee is not required per the ethics protocol since the study is open-label.

### ● The status and timeline of the study

The study is open in the recruiting phase. No interim analysis is currently planned.

## Discussion

### Rationale of the VA-HYPO study

Several authors have already investigated the effects of virtually augmented self-hypnosis (VA-HYPO) during medical procedures and found a significant improvement in patients’ experience of both anxiety and pain [19, 23–27]. A pilot study using commercially available software for VA-HYPO demonstrated good clinical outcomes on preoperative breast cancer patients [11] with both pain and anxiety reduction of about 40–60%. However, to our knowledge, there are no published data on VA-HYPO in the setting of percutaneous EVI.

Of note, patients’ experience in percutaneous image-guided therapies is not restricted to pain and anxiety that procedures could generate. Pain memory is also an essential determinant as patients might have multiple treatments with potentially multiple sessions in their life. Essentially, EVI requires puncturing one or more vessels with needles. Non-conscious memory of needle-induced pain can be shaped very early after the time of birth and influence subsequent responses to pain stimuli [28, 29]. Furthermore, pain memory in children has been found to be a predictor of future pain responses [30]. Interventions for pain memory-reframing might potentially affect distress and improve patients’ experience with EVI [31]. The device under evaluation in our study (Oncomfort^TM^) is lightweight, portable, and fast to set up and may be amenable to widespread clinical use with no additional human resources. Furthermore, the device can be controlled entirely by the operations in a sterile manner during the EVI procedure. The VA-HYPO trial aims to evaluate the impact of VA-HYPO on anxiety and pain during peripheral EVI, compared with peripheral EVI performed without VA-HYPO.

### Limitations of the study design

The clinical impact of the present study is potentially high, with improved patient wellbeing during peripheral EVI. We expect the results to be scientifically robust because of the prospective nature of the study, with randomization and following power analysis. The placebo effect could confound our results because of the impossibility of conducting a blinding procedure. However, we are interested in the net effect of virtually administered autohypnosis on perprocedural anxiety and pain; the placebo effect can contribute to a clinically significant improvement of procedural tolerance. Furthermore, past experiences with needle puncture were not assessed due to broad landscape covered not only by EVI, but any procedure performed under local anesthesia such as dental care, as well as subcutaneous or intramuscular injection or even surgery.

### Dissemination

Once terminated, we will submit the study results to a peer-reviewed scientific journal for publication. If significant enough, we will communicate the results to the public by means of scientific broadcasts.

We will implement substantial amendments only after approval by the external Ethics Committee.
